# Potentiated Activity of Amphotericin B-Loaded PLGA Nanoparticles Against *Aspergillus fumigatus*

**DOI:** 10.3390/polym18121421

**Published:** 2026-06-07

**Authors:** Anastasia Orekhova, Andrey V. Sybachin, Veronika Lyamina, Dana Mamiy, Alexander Osmolovskiy, Oxana M. Drapkina

**Affiliations:** 1 Biology Department, Lomonosov Moscow State University, Leninskie Gory 1-12, 119991 Moscow, Russia; lyaminavm@mail.ru (V.L.); danamamiy@yandex.ru (D.M.); aosmol@mail.ru (A.O.); 2 National Medical Research Centre for Therapy and Preventive Medicine, Ministry of Healthcare of the Russian Federation, Petroverigsky per. 10, Bld. 3, 101000 Moscow, Russia; drapkina@bk.ru; 3 Chemistry Department, Lomonosov Moscow State University, Leninskie Gory 1-3, 119991 Moscow, Russia

**Keywords:** *Aspergillus fumigatus*, *Galleria mellonella*, PLGA-NPs, amphotericin B, biofilm

## Abstract

Amphotericin B (AmB) is a broad-spectrum antifungal agent and a long-standing standard of care; however, its clinical use is compromised by poor solubility, off-target tissue distribution and severe dose-limiting toxicity. AmB-loaded poly (D, L-lactide-co-glycolide) nanoparticles (PLGA-AmB-NPs) were developed and characterized with respect to their physicochemical properties, antifungal activity against *A. fumigatus* biofilms, *in vivo* efficacy in the *Galleria mellonella* infection model, and hemolytic toxicity *in vitro*. Blank PLGA nanoparticles (PLGA-NPs) and PLGA-AmB-NPs exhibited mean diameters of 165 nm (PDI 0.075) and 120 nm (PDI 0.210), respectively, with negative zeta potential values consistent with colloidal stability in aqueous media. PLGA-AmB-NPs showed significantly enhanced activity against 24 h *A. fumigatus* biofilms compared with free AmB at concentrations of 5 and 10 µg/mL, while unloaded PLGA-NPs were inactive. Infected *G. mellonella* larvae treated with PLGA-AmB-NPs displayed markedly improved survival over a 5-day period relative to those receiving equivalent doses of free AmB. Furthermore, PLGA-AmB-NPs induced substantially lower hemolysis of human red blood cells than free AmB across all tested concentrations (5–20 µg/mL). PLGA-AmB-NPs represent a promising polymeric platform for the treatment of *A. fumigatus* infections.

## 1. Introduction

Fungal infections today represent one of the most alarming yet least addressed issues in global healthcare. The therapeutic arsenal is limited and outdated: there are fewer antifungal agents than antibiotics targeting other organisms, and the situation is further complicated by the fact that some of these drugs are toxic and have several drawbacks. Furthermore, resistance to some agents has already emerged. Thus, the development of non-toxic formulations of antifungal agents is highly relevant. Nanoparticle-based targeted drug delivery systems enhance bioavailability while simultaneously improving the capacity to overcome and penetrate biological barriers [1,2].

Amphotericin B (AmB) is an antifungal agent used for the treatment of mycoses caused by a wide range of pathogenic fungi, including *Candida* spp., *Aspergillus* spp., and *Mucorales* spp. [3]. AmB has remained a cornerstone of antifungal therapy for over six decades and is often described as the “gold standard” against a broad spectrum of life-threatening fungal infections [4]. However, in addition to acting on ergosterol in fungal cells, AmB also binds, albeit to a lesser extent, to cholesterol in kidney cell membranes, causing nephrotoxicity characterized by vasoconstriction and tubular damage [4,5,6]. In addition, conventional AmB is poorly soluble at physiological pH [3], induces the release of pro-inflammatory cytokines [5], distributes to non-target tissues [7], is degraded in the gastrointestinal tract and is not absorbed upon oral administration due to its hydrophobicity [8], exhibits limited penetration across the blood–brain barrier [9], and shows poor penetration into fungal biofilms [10].

Among AmB-susceptible pathogens, *A. fumigatus* is a leading cause of invasive aspergillosis in immunocompromised patients and is associated with high mortality rates [11]. In addition, *A. fumigatus* readily forms biofilms whose extracellular matrix limits antifungal penetration and confers increased tolerance to multiple drug classes, including polyenes [12]. Encapsulation in nanoparticles or lipid carriers helps to overcome the poor solubility of AmB and its limited delivery to “hard-to-reach” sites, reduces toxicity through more targeted distribution, and preserves drug integrity during transport to the site of action.

Currently, AmB is approved for use in several intravenous formulations [13], with its clinical application limited due to toxicity. Thus, modification of this promising antifungal agent is required to overcome these limitations. The novelty of this work lies in the combined evaluation of PLGA/PLA–PEG-based AmB nanoparticles across hemocompatibility, antibiofilm activity, and *in vivo* efficacy in an *A. fumigatus* infection model. The aim of this study was (1) to develop and characterize an AmB-loaded drug formulation using nanoparticles composed of poly(D,L-lactide-co-glycolide) (PLGA) and poly(D,L-lactide)-b-(ethylene glycol methyl ether) diblock-copolymer (PLA–PEG); (2) to investigate the hemolytic activity of the resulting formulation *in vitro*; (3) to evaluate the *in vitro* antifungal activity of PLGA-AmB-NPs against biofilm formation; and (4) to assess their therapeutic efficacy in *G. mellonella* larvae infected by *A. fumigatus*.

## 2. Materials and Methods

### 2.1. Reagents

Poly(D,L-lactide-co-glycolide) with average molecular weight Mw = 10,000 and a lactide to glycolide ratio of 50:50 (PLGA) and poly(D,L-lactide)-b-(ethylene glycol methyl ether) diblock copolymer (PLA–PEG) with PEG Mn = 2000 and PLA Mn = 2000 from Sigma-Aldrich (Saint Louis, MO, USA) were used as received. THF and methanol from Reachim (Moscow, Russia) and DMSO from Komponent-Reactiv (Moscow, Russia) were purified by distillation prior to the experiments.

### 2.2. Preparation and Characterization of PLGA-AmB-NPs

PLGA-NPs were synthesized according to the film hydration procedure described elsewhere [14] with the following modifications. A total of 10 mg of PLGA and 10 mg of PLA-PEG were placed in a round-bottom flask and dissolved in 2 mL of THF. Then, the solvent was evaporated using a Laborota 4000 vacuum rotary evaporator by Heidolph (Schwabach, Germany) at 40 °C. The resulting film was dispersed in 2 mL of DI water. Then the solution was exposed to ultrasound using a tip sonicator for 10 min with constant water cooling. As a result, a micellar suspension of PLGA-NPs was obtained.

PLGA-AmB-NPs were synthesized according to the procedure described above with the addition of AmB into the THF solution of polymers.

A methanol solution of AmB (75 mg/mL) was added to the polymer mixture to obtain a theoretical AmB weight fraction of 10 wt%, calculated as ω = mAmB/(mPLA − PEG + mPLGA + mAmB). As a result, a micellar solution of PLGA-AmB-NPs was obtained.

The characterization of PLGA-NPs and PLGA-AmB-NPs was performed by dynamic light scattering and zeta potential measurements using NanoBrook equipment by Brookhaven Industry Corporation (Holstville, NY, USA). The mean diameter and zeta potential of the particles were determined using the software Particle Solutions v4.0 supplied by the manufacturer.

#### Encapsulation Efficiency of AmB in PLGA-NP

**Figure 1:**
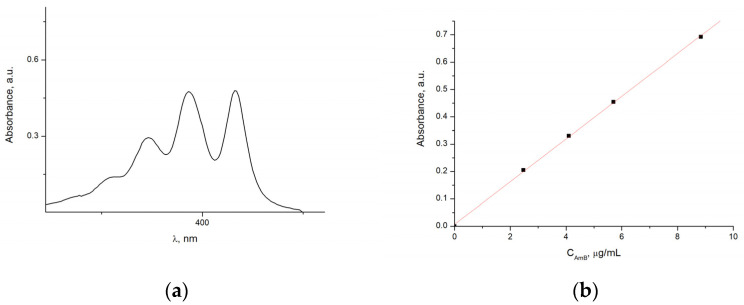
UV-vis spectrum of AmB in DMSO, concentration 5.7 µg/mL (**a**) and calibration curve for absorbance of AmB in DMSO at 416 nm (**b**).

The encapsulation efficiency (EE) was estimated by spectrophotometry using UV-vis spectrometer Ecros PE-5400UV (Ecroskhim, Saint-Petersburg, Russia) [15,16,17]. The PLGA-AmB-NPs suspension was subjected to centrifugation at 15,000 rpm on Eppendorf 5417C centrifuge (Eppendorf, Hamburg, Germany) to separate PLGA-AmB-Nps from unbound AmB. Supernatant aliquots were mixed with 3 mL of DMSO and the absorbance of the solution at 416 nm was registered. The calibration curve was obtained by measuring of AmB solutions in DMSO and was used to estimate the concentration of unbound AmB. A typical spectrum of AmB in DMSO and the calibration curve are presented in Figure 1.

The EE was calculated as follows:

$$EE=(\frac{CAmBtot-CAmBsup}{CAmBtot})\times 100\%$$

where C_AmB_^tot^- is the total concentration of AmB in the suspension, C_AmB_^sup^- is the concentration of AmB in the supernatant.

The resulting EE was found to be 99.0 ± 0.5%.

### 2.3. Fungal Strain and Culture Condition

*A. fumigatus* DSM 790, obtained from the German Collection of Microorganisms (DSMZ, Braunschweig, Germany), was used. The fungus was cultivated for five days on potato dextrose agar (Sigma-Aldrich, Saint Louis, MO, USA). The conidia were collected using phosphate-buffered saline (PBS) and counted with a hemocytometer before use. Tween 20 was excluded from the inoculum preparation to avoid possible interference.

### 2.4. Antifungal Susceptibility Testing

The minimum inhibitory concentration (MIC) of AmB, PLGA-AmB-NPs and PLGA-NPs against *A. fumigatus* DSM 790 was determined according to the standardized method (CLSI M38-A2 document) [18]. *A. fumigatus* DSM 790 was grown on potato dextrose agar (Sigma Aldrich, St. Louis, MI, USA) for 120 h. The concentration of the inoculum was 1.0 × 10^4^–2.5 × 10^4^ CFU/mL. *In vitro* antifungal activity was evaluated using concentrations ranging from 0.195 µg/mL to 100 µg/mL. The reported AmB concentrations for PLGA-AmB-NPs refer to the equivalent AmB concentration. Each experiment was performed at least three times, in triplicate, on separate dates. After 48 h, the MIC was calculated. After agitation of the plates, the growth in each well was compared with that of the growth control (drug-free) well with the aid of a reading mirror and a microplate reader (Thermo Multiskan EX, Thermo Fisher, Waltham, MA, USA).

### 2.5. Hemolytic Assay

Defibrinated human blood was obtained from de-identified healthy volunteers through the National Medical Research Center for Therapy and Preventive Medicine, Ministry of Healthcare (Moscow, Russia). Briefly, a 1 mL aliquot of blood was centrifuged at 1600× *g* for 10 min, after which the plasma was removed. The resulting red blood cell (RBC) pellet was washed at least three times with sterile 1× PBS, and following the final wash, the supernatant was discarded and the pellet was resuspended in 750 μL of sterile PBS. A 2% RBC suspension was then prepared by transferring 200 μL of the washed RBCs into a 15 mL conical tube containing 9.8 mL of sterile 1× PBS.

For the hemolysis assay, 50 μL aliquots of the 2% RBC suspension were dispensed into each well (*n* = 3) of a 96-well plate containing PLGA-NPs, PLGA-AmB-NPs, or free AmB at final concentrations ranging from 5 to 20 μg/mL. Wells containing 2% RBCs in 1× PBS alone served as the negative control, while 2% RBCs in 1% Triton X-100 served as the positive (100% hemolysis) control. The plate was incubated at 37 °C for 3 h and subsequently centrifuged at 1000× *g* for 2 min. The supernatant was carefully transferred to a fresh 96-well plate (tissue culture-treated, Falcon 353072, Corning Inc., Corning, NY, USA) and the absorbance was measured at 540 nm. The percentage of hemolysis was calculated relative to the Triton X-100 positive control as follows:

$$\%\;hemolysis=\frac{Abs\;sample-Abs\;negative\;control}{Abs\;positive\;control-Abs\;negative\;control}\times 100$$

All experiments were performed in triplicate [19]. A one-way ANOVA with Tukey’s post hoc test was used.

### 2.6. In Vitro Antifungal Activity of PLGA-AmB-NPs Against Biofilm Formation

The anti-biofilm activity was evaluated in 96-well plates as previously described. *A. fumigatus* DSM 790 was cultured on potato dextrose agar for five days. The conidia were harvested using PBS and counted with a hemocytometer. The inoculum was standardized to 1.0 × 10^6^ cells/mL. For biofilm formation assays, the compound concentrations ranged from 0.63 µg/mL to 10 µg/mL. After a 24 h incubation, the cells were washed, and the metabolic activity was assessed using the XTT reduction assay. An XTT-menadione was added, and the optical density at 450 nm was measured after incubation [20]. All experiments were conducted in triplicate on at least three separate dates. A one-way ANOVA with Tukey’s post hoc test was used.

### 2.7. In Vivo Activity of PLGA-AmB-NPs

To evaluate antifungal efficacy *in vivo*, sixth-instar *G. mellonella* larvae were infected via injection with *A. fumigatus* conidia (4 × 10^4^ to 5 × 10^4^ conidia per larva), followed by treatment with AmB, PLGA-AmB-NPs or PLGA-NPs. Control groups received sterile phosphate-buffered saline from Sigma-Aldrich (Saint Louis, MO, USA). Larval survival was recorded over a 120 h observation period. Mortality was assessed by visual inspection, with death defined as the concurrent presence of body discoloration (brown to dark brown) and unresponsiveness to mechanical stimulation with forceps [21]. All experiments were performed in triplicate. Survival data were analyzed by the Kaplan–Meier method with the Mantel–Cox log-rank test.

### 2.8. Toxicity of PLGA-NPs on Galleria mellonella Larvae Model

*In vivo* toxicity assessments were conducted using sixth-instar *G. mellonella* (Lepidoptera: Pyralidae) larvae (Life Moscow, Moscow, Russia), following previously established protocols. Larvae were maintained in wood shavings under dark conditions at 18 °C prior to experimentation. Only larvae weighing between 0.3 and 0.4 g were included; individuals exhibiting signs of melanization or discoloration (e.g., dark spots) were excluded from the study.

Either larvae were administered injections of AmB at varying concentrations, in free form or encapsulated within PLGA nanoparticles (PLGA-NPs), as well as blank PLGA-NPs. Control groups received either phosphate-buffered saline from Sigma-Aldrich (Saint Louis, MO, USA) or no injection. Survival was recorded over a 120 h observation period. Mortality was determined by visual inspection, with death defined as the combination of body discoloration (brown to dark brown) and absence of movement upon stimulation with forceps [21]. All experiments were performed in triplicate. Survival data were analyzed by the Kaplan–Meier method with the Mantel–Cox log-rank test.

### 2.9. Statistical Analysis

The data were expressed as mean ± SEM; *p* < 0.05 was considered statistically significant. Statistical criteria, *p*, and other parameters are shown for each experiment. The Kolmogorov–Smirnov test was applied to investigate the normality of the data distribution. *G. mellonella* survival was displayed via Kaplan–Meier curves. Statistical data analysis was performed using the GraphPad Prism 8 software (GraphPad Software Inc., La Jolla, CA, USA).

## 3. Results

### 3.1. Characterization of Nanoparticles

#### Size and Zeta Potential of NP

The sizes of NPs were determined in DI water. Representative intensity-weighted size distributions for blank and AmB-loaded PLGA nanoparticles are shown in Figure 1. The mean diameter of PLGA-NP was found to be 165 nm with a PDI value 0.075. The incorporation of AmB into the NPs did not dramatically change the sizes of the micelles. The mean diameter of the PLGA-AmB-NPs was found to be 120 nm with a PDI value 0.210. The broader polydispersity of the loaded micelles could be attributed to the disordering of the hydrophobic core of the PLGA-AmB-NPs by AmB molecules. However, the PDI value could be considered satisfactory in comparison with typical nanocarriers such as liposomes, microgels etc [22,23,24,25]. The measured zeta potential values (−15.5 ± 1.2 mV for PLGA-NP and −32.4 ± 1.4 mV for PLGA-AmB-NPs) indicate that the nanoparticles carry an overall net negative charge, consistent with their colloidal stability in aqueous media.

### 3.2. Antifungal Activity

#### 3.2.1. Activity Against *A. fumigatus* Planktonic Growth

The effects of AmB, PLGA-AmB-NPs, and PLGA-NPs on *A. fumigatus* planktonic growth were evaluated. AmB and PLGA-AmB-NPs demonstrated comparable inhibitory activity, with MIC_50_ and MIC_90_ values of 1 µg/mL and 2 µg/mL, respectively. Blank PLGA-NPs exhibited no measurable inhibitory activity against planktonic cells.

#### 3.2.2. Hemolytic Assay

The hemolytic activity of free AmB, PLGA-AmB-NPs, and PLGA-NPs was assessed against human erythrocytes across a concentration range of 5–20 µg/mL, encompassing concentrations up to 10-fold above the determined MIC_90_ (2 µg/mL). The percentage of hemolysis was calculated relative to the Triton X-100 positive control, defined as 100% hemolysis.

**Figure 2:**
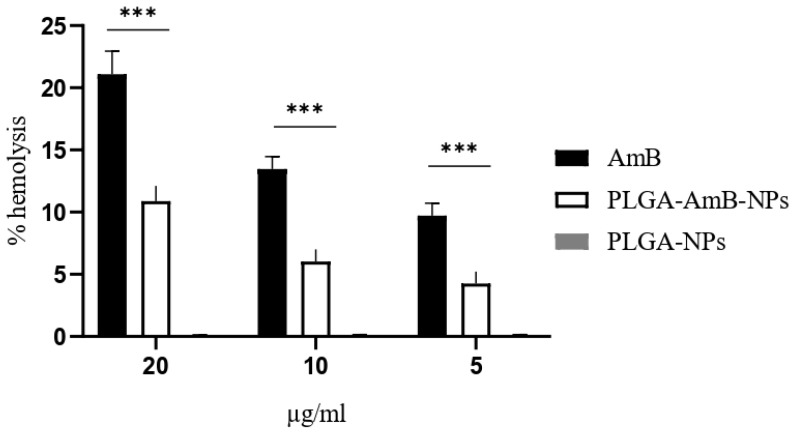
*In vitro* hemolytic activity of free AmB, PLGA-AmB-NPs and PLGA-NPs at varying concentrations. It was not detected hemolytic activity PLGA-NPs. Data are expressed as mean ± SD (*n* = 3), *** *p* < 0.001 for PLGA-AmB-NPs vs. free AmB (one-way ANOVA with Tukey’s post hoc test).

At all tested concentrations, PLGA-AmB-NPs exhibited significantly lower hemolytic activity compared to free AmB (*p* < 0.001), Figure 2. Unloaded PLGA-NPs exhibited no hemolysis effect. These findings indicate that the encapsulation of AmB within PLGA nanoparticles substantially reduces its hemolytic potential across all tested concentrations, suggesting improved safety of PLGA-AmB-NPs compared to the free drug.

#### 3.2.3. *In Vitro* Antifungal Activity of PLGA-AmB-NPs Against Biofilm Formation

**Figure 3:**
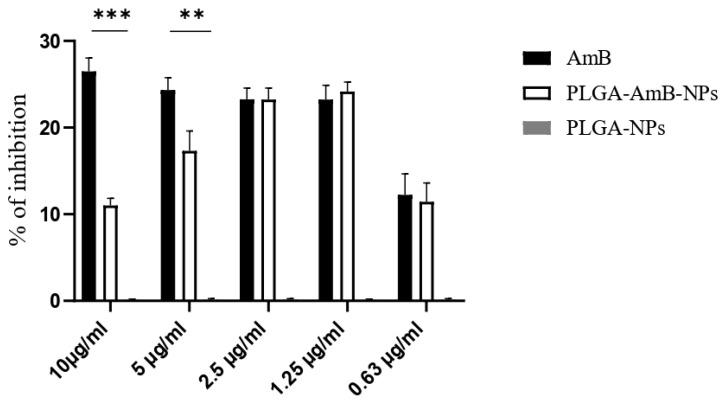
Activity of free AmB, PLGA-AmB-NPs and PLGA-NPs against 24 h *A. fumigatus* biofilm. Antifungal activity of PLGA-NPs was not detected against 24 h *A. fumigatus* biofilm. ** *p* < 0.01, *** *p* < 0.001 for PLGA-AmB-NPs vs. free AmB (one-way ANOVA with Tukey’s post hoc test).

The antifungal efficacy of free AmB and PLGA-AmB-NPs against biofilm formation was assessed *in vitro* by measuring the metabolic activity of the fungal cells. Following a 24 h of incubation, PLGA-AmB-NPs demonstrated significantly superior antifungal activity compared to free AmB at concentrations of 10 µg/mL and 5 µg/mL (Figure 3). Unloaded PLGA-NPs exhibited no antifungal effect.

#### 3.2.4. Activity of PLGA-AmB-NPs, Free AmB and PLGA-NPs on *G. mellonella* Larvae Model

**Figure 4:**
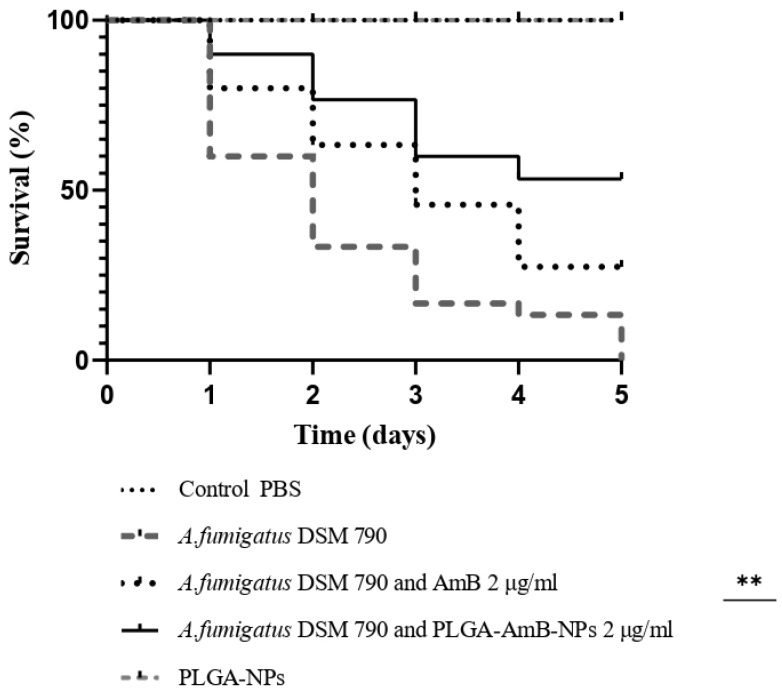
PLGA-encapsulated AmB exhibited significantly superior antifungal efficacy against *A. fumigatus* virulence in the *G. mellonella* wax moth model compared to free AmB. Survival of *G. mellonella* larvae (*n* = 30 per group) was monitored over a 5-day period following injection with 4 × 10^4^ to 5 × 10^4^ conidia per larva of *A. fumigatus*. Statistical differences relative to the PBS control were assessed using the Kaplan–Meier method with Mantel–Cox log-rank test, where ** *p* < 0.01 denote significant differences compared to the *A. fumigatus* and AmB 2 µg/mL and *A. fumigatus* and PLGA-AmB-NPs 2 µg/mL. All results represent data from a minimum of three independent biological replicates.

The *in vivo* efficacy of the formulations was assessed using the *G. mellonella* infection model, a well-established surrogate system for studying fungal virulence owing to the functional homology between its innate immune responses and those of vertebrates. Larvae were inoculated with *A. fumigatus* conidia at a dose of 4–5 × 10^4^ conidia per larva and subsequently treated with free AmB or PLGA-AmB-NPs at a concentration of 2 µg/mL, corresponding to the MIC_90_. Larval survival was recorded daily over a 5-day observation period. Larvae administered PBS or PLGA-NPs alone served as negative controls and exhibited 0% mortality, whereas all larvae infected with *A. fumigatus* conidia succumbed by day 5 post-infection, corresponding to 100% mortality. Notably, AmB encapsulated within PLGA-NPs demonstrated significantly better antifungal activity compared to free AmB (*p* < 0.01), Figure 4.

#### 3.2.5. *In Vivo* Toxicity Assessment in the *G. mellonella* Larva Model

PLGA-NPs, PLGA-AmB-NPs and AmB were tested in an *in vivo* toxicity model. The LD_50_ detected dose was more than 500 mg/kg for AmB and more than 800 mg/kg for PLGA-AmB-NPs (Table 1).

## 4. Discussion and Conclusions

Nanoparticles represent a promising pharmaceutical strategy for antifungal drug delivery owing to their small size, which enables penetration into capillaries and facilitates efficient accumulation at target sites [26,27]. The development of AmB-loaded PLGA nanoparticles has progressed substantially over the past decades. Early work by Venier-Julienne et al. [28] using the solvent evaporation method resulted in very low drug loading (0.7–1.3%) due to poor miscibility between PLGA and AmB. Subsequent advances, including nanoprecipitation techniques stabilized with vitamin E TPGS, enabled improved particle size control and drug incorporation [29]. These advanced systems achieved dual benefits: reduced toxicity compared to conventional formulations such as Fungizone^®^, and superior therapeutic efficacy in murine aspergillosis models, demonstrating performance comparable to or exceeding that of parenteral liposomal formulations such as AmBisome^®^ in oral delivery studies [30].

The physicochemical characterization in this study revealed that the blank PLGA NPs had a mean diameter of 165 nm with a low polydispersity index (PDI 0.075), whereas the AmB-loaded nanoparticles exhibited a smaller mean size (~120 nm) with a moderately broader distribution (PDI 0.210). Both formulations displayed a net negative surface charge, with zeta potentials of −15.5 ± 1.2 mV (blank NPs) and −32.4 ± 1.4 mV (AmB-loaded NPs), indicating good colloidal stability. The drug loading efficiency was found to be 99.0+/−0.5%. These values are consistent with previously reported PLGA-based antifungal systems, which typically fall within a size range of 50–250 nm and exhibit ζ-potentials between −20 and −30 mV [31,32]. The observed decrease in particle size and increased surface charge following drug encapsulation likely reflect structural reorganization within the nanoparticle core and surface composition.

PLGA-AmB-NPs reduced the metabolic activity of biofilm-embedded cells more effectively than free AmB at equivalent concentrations (5 and 10 µg/mL), while blank NPs showed no antifungal activity against *A. fumigatus* biofilms *in vitro*. This improvement may be attributed to the enhanced penetration of the nanoparticles through the extracellular biofilm matrix and sustained drug release at the infection site. Similar findings have been reported for other PLGA-based antifungal systems, including PLGA-PTB nanoparticles targeting *Aspergillus* biofilms [32]. Additionally, Yang et al. [33] demonstrated that AmB-loaded PLGA nanoparticles combined with ultrasound significantly disrupted *C. albicans* biofilms, reducing biomass and enzymatic activity while altering biofilm structure, suggesting that nanoparticle-based strategies can be further enhanced through combinatorial approaches.

*In vivo* efficacy was confirmed using the *G. mellonella* infection model, in which larvae treated with PLGA-AmB-NPs demonstrated significantly improved survival relative to those receiving equivalent doses of free AmB. These findings are in agreement with previous studies employing PLGA-based formulations in similar infection models [31], as well as mammalian studies demonstrating enhanced therapeutic outcomes and reduced toxicity [29,34]. For example, Souza et al. [34] reported significant antifungal activity of PLGA-DMSA AmB nanoparticles at relatively low doses without observable toxicity, while Tang et al. [35] developed targeted, pH-responsive PLGA-based nanoparticles that improved antifungal efficacy and reduced toxicity in both *in vitro* and *in vivo* systems.

A major advantage of PLGA-based nanoformulations is the reduction in AmB-associated toxicity. In the present study, amphotericin B-loaded PLGA nanoparticles were successfully developed and demonstrated reduced hemolytic toxicity compared with the free drug. The dose-dependent hemolysis observed for both formulations aligns with the known mechanism of AmB-induced membrane damage via interaction with cholesterol in erythrocyte membranes [34,36]. These findings are consistent with earlier reports, particularly those of Italia et al. [29,37], who showed that PLGA-based AmB nanoparticles significantly decreased hemolytic activity and renal toxicity while achieving oral bioavailability as high as 800%, highlighting their promise for oral delivery in systemic fungal infections.

Despite these promising findings, several limitations should be acknowledged. The current study evaluated only a single *A. fumigatus* strain and focused on early-stage biofilm formation; thus, the efficacy of PLGA-AmB-NPs against mature biofilms and diverse clinical isolates, including azole-resistant strains, remains to be determined. Additionally, the pharmacokinetics and biodistribution were not assessed, which are critical parameters in comparison with clinically approved lipid formulations. While the *G. mellonella* model provides valuable preliminary insights, validation in mammalian models is essential before clinical translation. Future studies should therefore focus on optimizing nanoparticle formulations, evaluating *in vivo* pharmacokinetics, and exploring combination therapies or targeting strategies to further enhance antifungal efficacy and safety. These results indicate that PLGA-based nanoencapsulation can increase the therapeutic index of AmB by simultaneously potentiating its antifungal efficacy and mitigating its toxicity. PLGA-AmB-NPs therefore represent a promising polymeric platform for the treatment of *A. fumigatus* infections, particularly those involving biofilm-associated growth, and warrant further investigation in extended strain panels, azole-resistant isolates and mammalian models of aspergillosis.

## Figures and Tables

**Table 1:** Survival of *G. mellonella* larvae following administration of PLGA-NPs, PLGA-AmB-NPs by intra-haemocoel injection. Each experiment was conducted in triplicate with 10 larvae per group. All values are the mean of three independent experiments. Data were analyzed by the Kaplan–Meier method with the Mantel–Cox log-rank test.

Chemical	LD_50_ (mg/kg)	Solvent
PLGA-NPs	-	H_2_O
AmB	>500	100 H_2_O:1 DMSO
PLGA-AmB-NPs	>800	100 H_2_O:1 DMSO

## Data Availability

The original contributions presented in this study are included in the article. Further inquiries can be directed to the corresponding author.
